# Optical Resolution of Two Pharmaceutical Bases with Various Uses of Tartaric Acid Derivatives and Their Sodium Salts: Racemic Ephedrine and Chloramphenicol Base

**DOI:** 10.3390/molecules27103134

**Published:** 2022-05-13

**Authors:** Dorottya Fruzsina Bánhegyi, Elemér Fogassy, János Madarász, Emese Pálovics

**Affiliations:** 1 Department of Organic Chemistry and Technology, Budapest University of Technology and Economics, Műegyetem rkp. 3, H-1111 Budapest, Hungary; banhegyi.dorottya.fruzsina@vbk.bme.hu (D.F.B.); fogassy.elemer@vbk.bme.hu (E.F.); 2 Department of Inorganic and Analytical Chemistry, Budapest University of Technology and Economics, Műegyetem rkp. 3, H-1111 Budapest, Hungary; madarasz.janos@vbk.bme.hu

**Keywords:** optical resolution, diastereomeric salt formation, enantiomeric separation, crystallographic unit cell

## Abstract

The optically active dibenzoyltartaric acid, tartaric acid, and its sodium salts were successfully applied to the optical resolution of (1*R*,2*S*)(1*S*,2*R*)-2-(methylamino)-1-phenylpropan-1-ol (**EPH**) and (1*R*,2*R*)(1*S*,2*S*)-2-amino-1-(4-nitrophenyl)propane-1,3-diol (**AD**) as resolving agents. It was observed that both compounds’ resolution using a mixture of salts of quasi-racemic resolving agents showed a change in chiral recognition under the same conditions compared to the result of the use of the single enantiomeric resolving agent. The changes are followed by detailed analytical (XRD, FTIR, and DSC) studies. Meanwhile, the DASH indexing software package was also tested on powder XRD patterns of pure initial materials and intermediate salt samples of high diastereomeric excess.

## 1. Introduction

The pharmaceutical industry has always required the preparation of pure enantiomeric form; the most straightforward way, besides the countless enantioselective methods, is a resolution based on diastereomeric salt formation [1,2,3,4,5]. This classical formulation of resolution is robust, well scalable, and is the simplest way to separate acidic or basic character compounds with a resolving agent of an opposite character. By providing suitable conditions, the diastereomers can be forced to divide between two phases and separate from each other. This paper presents “half-equivalent” methods for resolution via the diastereomeric salt formation of two racemic pharmaceutical intermediates with a basic character.

Several studies on the optical resolution of racemic ephedrine are reported. As contained in the literature, the resolving agents used for resolving DL-ephedrine include L-monomenthol ester of succinic acid [6], D- and L-mandelic acid [7], D-α-[4-arsono-anilino]-propionamide, D-arabonic acid, D-tartaric acid in methanol, and in aliphatic alcohols having 2 to 8 carbons [8].

Racemic aminodiol (**AD**), i.e., 2-amino-1-(4-nitrophenyl)propane-1,3-diol, is a central intermediate in chloramphenicol (Chlorocide) production in the pharmaceutical industry, protected by more than 14 patents, the active ingredient in medicines from more than 23 brands [9,10]. Since chloramphenicol is already a chiral molecule, (1*R*,2*R*)-(1-*p*-nitrophenyl-2-dichloroacetamido-1,3-propanediol), a resolution of racemic **AD** is key in the synthesis. This article aims to summarize our experiments by successfully applying dibenzoyltartaric acid and tartaric acid Na^+^-salts as the resolving agents in resolving the two bases starting from their racemic HCl salts.

## 2. Results and Discussion

### 2.1. Resolution of Ephedrine·HCl with Half-Equivalent of Resolving Agent

**Figure 1:**
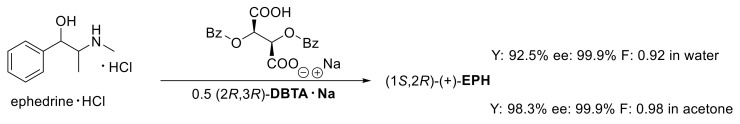
Optical resolution with half-equivalent of (2*R*,3*R*)-**DBTA·Na**.

The resolution of ephedrine·HCl was attempted in several ways. Racemic ephedrine hydrochloride is resolved with half an equivalent of (2*R*,3*R*)-**DBTA·Na**. The result of the resolution is often influenced by the solvent [11], so the resolutions were also performed in acetone and water. The diastereomeric salt was allowed to crystallize for 2 h, and the pure crystalline enantiomer was isolated in 92.5% yield (Figure 1). In each case, the yields of the pure enantiomers were compared to half of the weight of the racemic compound.

### 2.2. Resolution of Ephedrine·HCl with Quasi-Enantiomeric Resolving Agent Mixture

**Figure 2:**
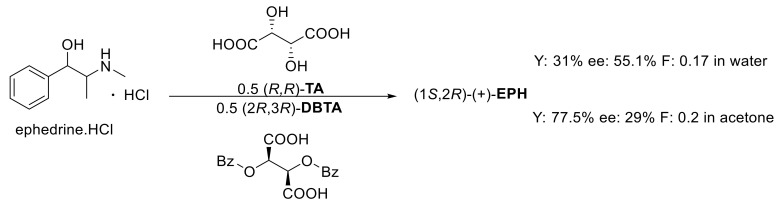
Optical resolution with the quasi-enantiomeric resolving agent mixture.

Our research group previously investigated the use of quasi-racemic resolving agents in the separation of tofisopam [12], where better separation could be achieved compared to the half-equivalent resolving agent initially used. The application of a quasi-enantiomeric resolving agent resulted in a yield of 31% and enantiomeric purity of 55% (F: 0.17, Figure 2).

### 2.3. Resolution of Ephedrine·HCl with the Sodium Salts of the Quasi-Enantiomeric Resolving Agent Mixture

**Figure 3:**
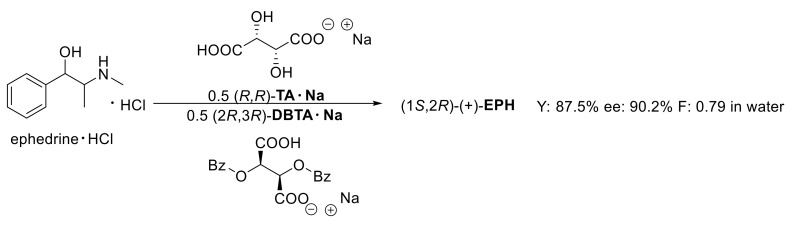
Optical resolution with the sodium salts of the quasi-enantiomeric resolving agent mixture.

By preparing sodium salts of the resolving agent, we used ((2*R*,3*R*)-**DBTA·Na** and (*R*,*R*)-**TA·Na** and then performed the resolution, the ((1*S*,2*R*)-(+)-**EPH**) was enriched in the diastereomeric salt. The decomposition of the precipitated diastereomeric salt with aqueous ammonia yielded the precipitated (1*S*,2*R*)-(+)-**EPH**: 87.5%, ee: 90% and F: 0.79 (Figure 3).

$$Y_{enant.}=\frac{m_{enant.}}{\left(m_{rac.}\right)/2}\times 100\%$$

### 2.4. Resolution of Aminodiol with the Sodium Salt of (S,S)-DBTA

**Figure 4:**
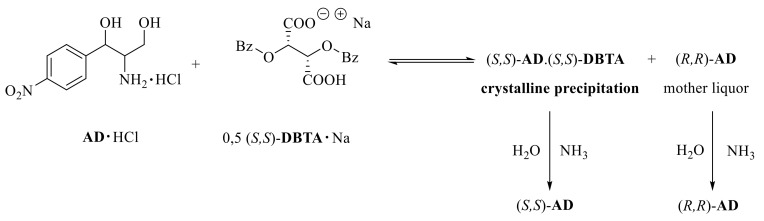
Resolution of **AD** with (*S*,*S*)-**DBTA·Na**.

The resolution of the racemic **AD·HCl** salt was started with (*S*,*S*)-**DBTA·Na**. An aqueous solution of 0.5 equivalents of NaOH and 0.5 equivalents of (*S*,*S*)-**DBTA** was prepared, followed by adding a stock solution of the **AD·HCl** salt (Figure 4). The mixture was then allowed to crystallize, and after filtration, the obtained diastereomer was decomposed with aqueous ammonia. The enantiomer in the mother liqor was also recovered after adding aqueous ammonia.

**Figure 5:**
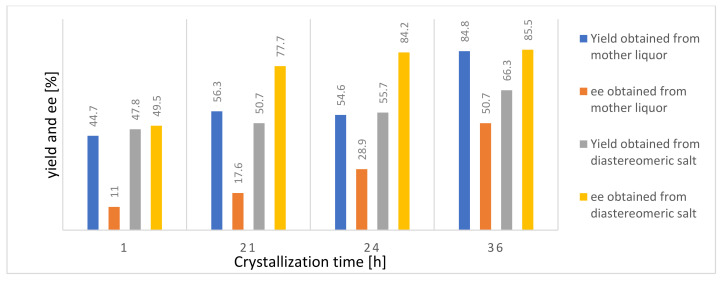
Effect of crystallization time on yield and enantiomeric purity measured from the enantiomers obtained from the (*S*,*S*)-**AD**·(*S*,*S*)-**DBTA** diastereomeric salt and from the filtered mother liqor.

It can be seen in Figure 5 that the yield of the (*S*,*S*)-**AD** enantiomers obtained from the diastereomeric salt increased with time: 47.8% after 1 h of crystallization and 66.3% after 36 h. In parallel, the enantiomeric purity also increased, but there was no longer a big difference between the 24 and 36 h results (Table 1) since the thermodynamic equilibrium was established by this time. Based on this, it can be seen that it is more appropriate to use a longer reaction time because the effect of thermodynamic control dominates during the resolution.

According to Figure 5, the highest yield of the enantiomer recovered from the mother liqor (84.8%) was observed at the 36 h resolution time. The enantiomer was continuously purified over time; therefore, a longer reaction time is beneficial. The best separation was achieved with a crystallization time of 36 h, and in this case, thermodynamic control prevailed during the resolution.

### 2.5. Resolution of Aminodiol with Sodium Salts of the Quasi-Racemic Resolving Agent Mixture of (S,S)-**DBTA** and (R,R)-**TA**

Resolving the racemic **AD·HCl** salt was subsequently attempted with a quasi-racemic resolving agent mixture, as Dutch researchers [13,14,15] recognized that in some cases, a better separation could be achieved with a related molecular structured resolving agent mixture than using the resolving agents alone.

**Figure 6:**
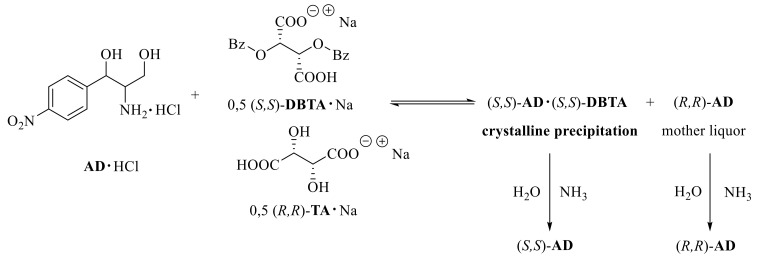
Resolution of Aminodiol with a mixture of quasi-racemic resolution agents.

First, an aqueous solution of 0.5 equivalents of NaOH and 0.5 equivalents of (*R*,*R*)-**TA** was prepared by adding a stock solution of the **AD·HCl** salt. An aqueous solution of 0.5 equivalents of NaOH and 0.5 equivalents of (*S*,*S*)-**DBTA** was then prepared and poured onto the previous solution. The mixture was then allowed to crystallize. After filtration of the precipitated diastereomer, it was decomposed with aqueous ammonia (Figure 6). The enantiomer in the mother liqor was also precipitated in the same way.

The yield of the enantiomer obtained from the diastereomeric salt decreased over time: 71.2% after 1 h and 52.2% after 36 h. In parallel, the enantiomeric purity decreased drastically: 97.7% after 1 h and 64.5% after 36 h (Table 2). Based on this, it can be seen that a longer reaction time for this resolution was not advantageous since the effect of the kinetic control prevails here.

The best yield of the enantiomer obtained from the mother liqor (64.6%) was observed at the crystallization time of 1 h. The purity of the enantiomer decreased with time, so these results also show the dominance of the kinetic control.

## 3. Materials and Methods

### 3.1. Optical Resolution

The enantiomeric purity of enantiomeric mixtures is determined by their optical rotation, which was determined on a Perkin-Elmer 241 polarimeter.

#### 3.1.1. Resolution of Ephedrine·HCl with Half-Equivalent Amount of Resolving Agent

To a mixture of 0.20 g (1 mmol) of racemic ephedrine·HCl and 0.04 g (0.5 mmol) of NaOH, 0.18 g (0.5 mmol) of (*R,R*)-(+)-**DBTA** were added to 1.5 mL of water, which was heated until dissolved. After cooling and standing for 120 min, the crystalline precipitate was filtered off. The diastereomeric salt thus obtained (0.26 g) was suspended in 0.5 mL of water, and 0.2 mL of NH_4_OH was added to the mixture.

The weight of (1*S*,2*R*)-(+)-ephedrine obtained was 0.074 g, ${\left[\alpha \right]}_{D}^{20}$ = +43.2 (c = 1, MeOH), Y: 92.5%, ee ~100%, F: 0.92.

The mother liqor was decomposed with 0.4 mL of NH_4_OH, but no precipitate was observed.

#### 3.1.2. Resolution of Ephedrine·HCl with Quasi-Enantiomeric Resolving Agent Mixture

To a mixture of 0.20 g (1 mmol) of racemic ephedrine.HCl, 0.04 g (0.5 mmol) of NaOH, 0.18 g (0.5 mmol) of (2*R*,3*R*)-(+)-**DBTA**, 0.08 g (*R*,*R*)-(+)-**TA** (0.5 mmol) were added 1.5 mL acetone and heated to dissolution. After cooling, the crystalline precipitate was immediately filtered. The diastereomeric salt thus obtained (0.16 g) was suspended in 0.5 mL of water, and 0.2 mL of NH_4_OH was added to the mixture.

The weight of (1*S*,2*R*)-(+)-ephedrine obtained was 0.062 g, ${\left[\alpha \right]}_{D}^{20}$ = +11.59 (c = 1, MeOH), Y: 77.5%, ee: 29%, F: 0.2.

The resolution was also performed in 5 mL water, the weight of (1*S*,2*R*)-(+)-ephedrine obtained was 0.054 g, ${\left[\alpha \right]}_{D}^{20}$ = +2.6 (c = 1, MeOH), ee: 55.1%, Y: 31%, F: 0.17.

#### 3.1.3. Resolution of Ephedrine·HCl with the Sodium Salt of a Quasi-Enantiomeric Resolving Agent Mixture

To the mixture of 0.40 g (2 mmol) of racemic ephedrine.HCl and 0.17 g (1 mmol) of (*R*,*R*)-(+)-**TA·Na,** 0.08 g (1 mmol) of (*R*,*R*)-(+)-**DBTA·Na** salt were added to 2.5 mL water and then heated until dissolved. After cooling, the crystalline precipitate was immediately filtered. The diastereomeric salt thus obtained (0.48 g) was suspended in 0.5 mL of water, and 0.4 mL of NH_4_OH was added.

The weight of (1*S*,2*R*)-(+)-ephedrine obtained 0.14 g, ${\left[\alpha \right]}_{D}^{20}$ = +37.4 (c = 1, MeOH), Y: 87.5%, ee: 90%, F: 0.79.

#### 3.1.4. Resolution of Aminodiol with the Sodium Salt of (*S*,*S*)-**DBTA**

NaOH (0.35 g, 8.75 mmol) and *(S*,*S*)-**DBTA** (3.13 g, 8.75 mmol) were dissolved in 10 mL of distilled water and added to 30 mL of stock solution of racemic **AD·HCl** (containing: 4.35 g, i.e., 17.5 mmol). The mixture was shaken and then resolved with different crystallization times.

The precipitated diastereomeric salt was filtered off, washed twice with a bit of water, and then concentrated NH_4_OH was added with a mass equal to 2× the wet weight. After 2 h of crystallization, it was filtered and dried.

The other **AD** enantiomer was precipitated from the mother liqor with concentrated NH_4_OH with a mass equal to 3× the weight of the wet diastereomeric salt. After 20 h of crystallization, the enantiomer was filtered and dried.

#### 3.1.5. Resolution of Racemic Aminodiol with a Quasi-Racemic Resolving Agent Mixture of (*S*,*S*)-**DBTA** and (*R*,*R*)-**TA** Salts

A total of 0.35 g (8.75 mmol) of NaOH and 1.31 g (8.75 mmol) of (*R*,*R*)-**TA** were combined and dissolved in 5 mL of distilled water, to which 30 mL of the stock solution of racemic **AD·HCl** (containing: 4.35 g, i.e., 17.5 mmol) was added. NaOH (0.35 g, 8.75 mmol) and (*S*,*S*)-**DBTA** (3.13 g, 8.75 mmol) were dissolved in distilled water (10 mL), and the two solutions were combined. The mixture was shaken and then resolved with different crystallization times.

Then, the precipitated diastereomeric salt was filtered, washed twice with a bit of water, and then concentrated NH_4_OH was added with a mass equal to 4× the wet weight. After 2 h of crystallization, the enantiomer was filtered and dried.

The other **AD** enantiomer was precipitated from the mother liqor with concentrated NH_4_OH with a mass equal to 6× the weight of the wet diastereomeric salt. After 20 h of crystallization, the enantiomer was filtered and dried.

### 3.2. Analytical Characterization of Starting Materials to Be Resolved and Intermediate Diastereomeric Salts of Resolutions

In order to figure out some possible reasons for favorable mechanisms and overall resolution processes, a detailed analytical study on all the initial materials involved and intermediate diastereomeric solid samples obtained have been carried out by powder X-ray diffraction (XRD), FT-IR spectroscopy, and differential scanning calorimetry (DSC), see below in detail.

Meanwhile, the application range of the powder XRD pattern-indexing and crystal structure modelling/solving opportunities connected to the DASH software package [16] have been also tested on pure initial materials and diastereomeric salt samples of high diastereomeric excess [17].

#### 3.2.1. Experimental Methods of Characterization

##### Powder X-ray Diffraction (XRD)

Powder XRD patterns were recorded with an X’pert Pro MPD (PANalytical B.v., Almelo, The Netherlands) multipurpose X-ray diffractometer using Cu Kα radiation with a Ni filter, X’celerator detector, and “zero background” single crystal silicon or “top-loaded” sample holders in the range of 2*θ* = 4–44°. The X-ray tube was operating at 40 kV and 30 mA. For purposes of indexing and simulated annealing structure solution by DASH [16] software package, a step size of 0.0167° up to 2*θ* = 52° was applied.

##### FT-IR Spectroscopy

Fourier transform infrared spectra of the solid powdered samples were measured using a PE System 2000 (Perkin Elmer) FTIR spectrophotometer in KBr between 500 and 4000 cm^−1^.

##### Differential Scanning Calorimetry (DSC)

DSC measurements were performed in a DSC 2920 device (TA Instruments Inc., New Castle, DE, USA). The powdered samples (ca. 3.5 mg) were measured in hermetically sealed Al pans, applying a heating rate of 10 K/min.

Measurement of N-content of **(1*S*,2*R*)-(+)-Ephedrinium** **(2*R*,3*R*)-dibenzoyl-bitartrate** (1:1) salt labelled as ‘**I, EPH-DBTA (1:1)**’, was carried out according to Dumas method by an FP-528 (LECO) apparatus in 3 parallel measurements: 2.88, 2.87, and 2.86%, (calculated theoretical nitrogen content for salts with EPH:DBTA = 1:1 or 2:1 molar ratio would be 2.675 or 4.067 N%, respectively).

**Figure 7:**
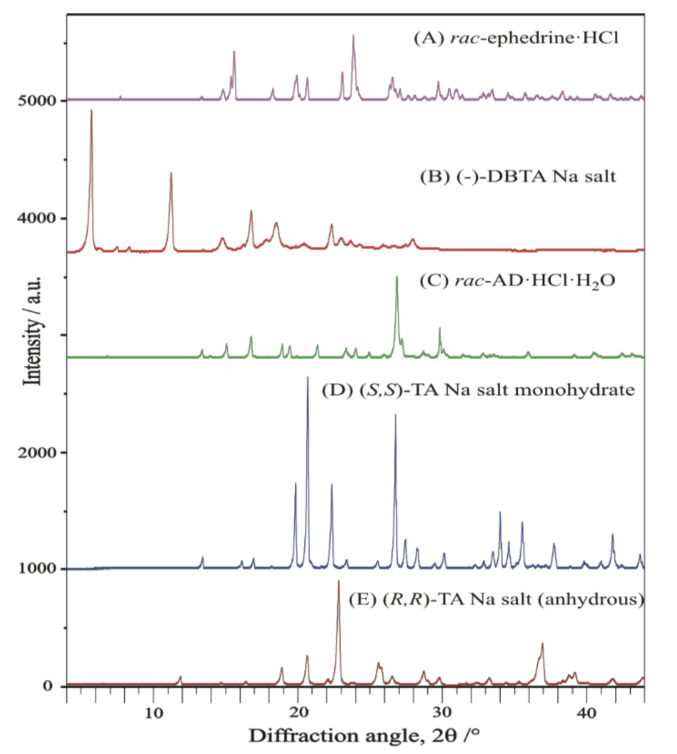
Powder XRD profile of starting materials: (**A**) racemic ephedrine hydrochloride ‘***rac*-EPH·HCl**’; (**B**) racemic Chloramphenicol base hydrochloride monohydrate ‘***rac*-AD·HCl·H_2_O**’; (**C**) (-)-sodium-dibenzoyl-bitartrate salt **(-)-DBBS-Na**; (**D**) sodium (*S*,*S*)-bitartrate salt monohydrate, **(*S*,*S*)-BS-Na·H_2_O**; (**E**) anhydrous sodium (*R*,*R*)-bitartrate salt **(*R*,*R*)-BS-Na**.

**Figure 8:**
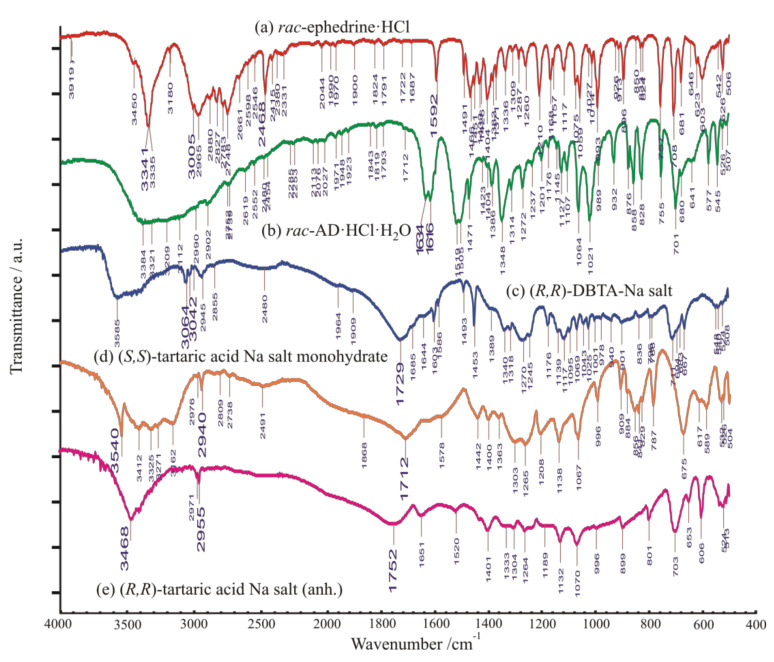
FT-IR spectra of starting materials: (**a**) racemic ephedrine hydrochloride ‘***rac*-EPH·HCl**’; (**b**) racemic Chloramphenicolbase hydrochloride monohydrate ‘***rac*-AD·HCl·H_2_O**’; (**c**) (-)-sodium-dibenzoyl-bitartrate salt ‘**(-)-DBBS-Na**’; (**d**) sodium (*S,S*)-bitartrate salt monohydrate ‘**(*S*,*S*)-BS-Na·H_2_O**’; (**e**) anhydrous sodium (*R*,*R*)-bitartrate salt ‘**(*R*,*R*)-BS-Na**’.

Characteristic XRD profiles and corresponding FTIR spectra of the starting materials, applied in all the resolution processes, are shown in comparison in Figure 7 and Figure 8, respectively.

#### 3.2.2. Identification of Solid Starting Materials

##### Identification of Solid Racemic Ephedrine Hydrochloride to Be Resolved

**Figure 9:**
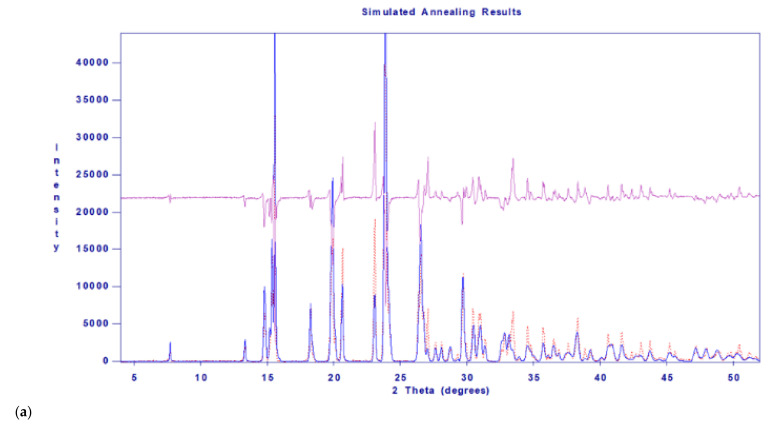

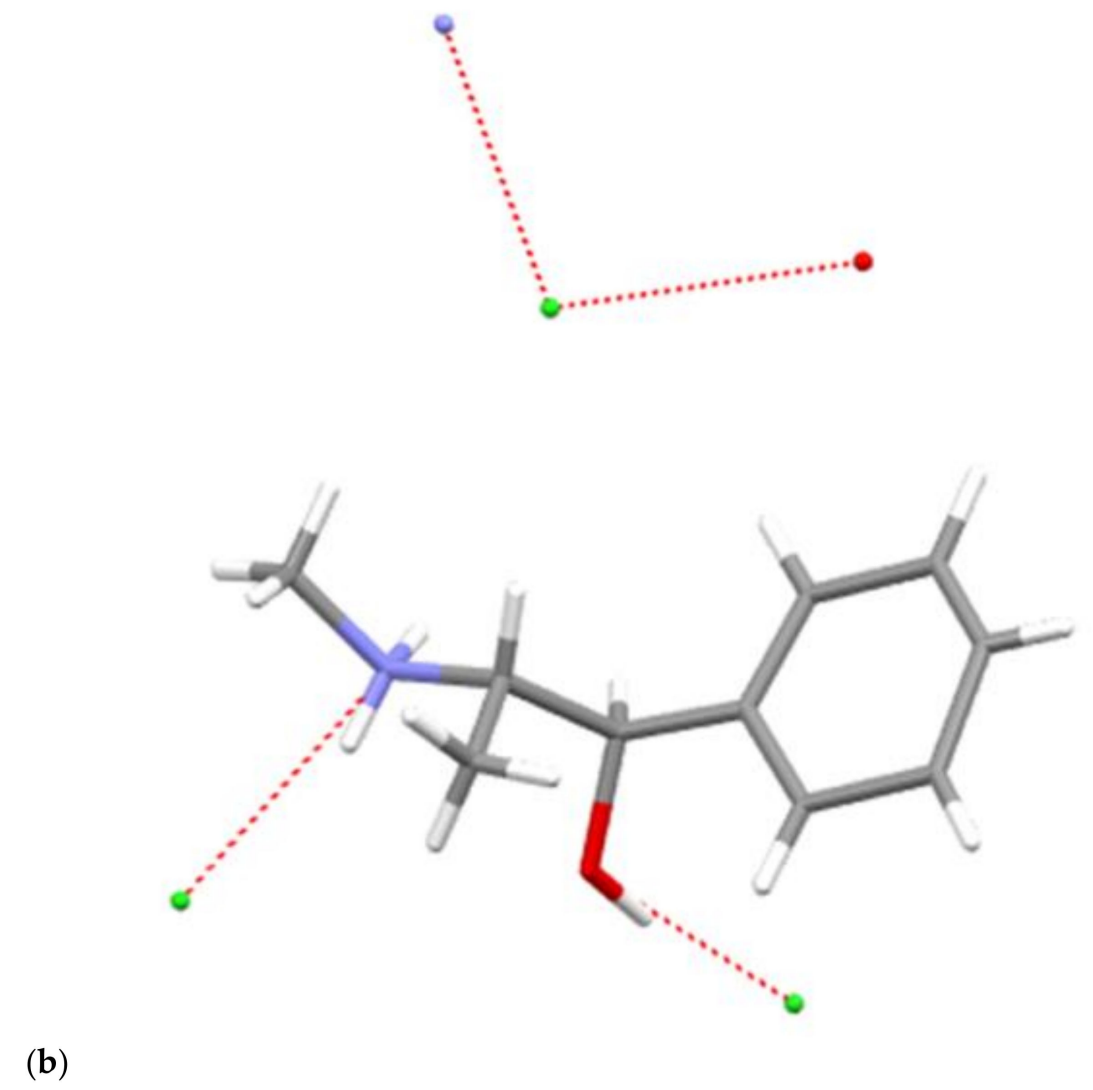
(**a**) Profile fitting and (**b**) unit cell contents and hydrogen bonds achieved with the DASH program package [16] for the ‘*rac*-**EPH·HCl**’ sample to be resolved.

The racemic ephedrine hydrochloride sample being resolved, labelled as ‘***rac***-**EPH·HCl**’, was identified by its powder XRD profile, see Figure 7A. Its profile was found to match that of the reference pattern of the DL-Ephedrine hydrochloride substance, PDF File No. 00-032-1675 [18], found in the international Powder Diffraction File (PDF-4+, Release 2021) [19] database. Although the unit cell of this substance has been of crystallographic interest since 1933 [20], there is no structure with 3D atomic coordinates available of it in the Cambridge Structural Database [21] (CSD [22]) yet. Based on the measured powder XRD profile of the sample, we have carried out a structure solution, like before in [23]. The estimated unit cell parameters are summarized in Table 3 in comparison with previous references. While profile fitting, asymmetric unit cell contents, and hydrogen bridges achieved with the DASH program package [16] are shown in Figure 9a,b. A skeleton of the solved structure of enantiomeric ephedrine hydrochloride (CSD code EPHECL02 [24]) with known bond distances and angles was applied as a model for ammonium ion, but with a rigid-body refinement on torsion angles. All the solved enantiomeric crystal structures in the CSD database (EPHECL [25], EPHECL01 [26], EPHECL02 [24], and EPHECL05 [27]) exhibit a bit more complex hydrogen bonds system (involving all NH_2_ and even OH protons) than the single N-H … Cl and O-H … Cl hydrogen bonds obtained in our structural trial for the racemic salt (Figure 9b). Anyhow, the enantiomeric hydrochloride with three hydrogen bonds had a higher melting point (219.0 °C) and higher thermodynamic stability than that of the racemic one (190.8 °C, [28]) with one or two hydrogen bonds. The FT-IR spectrum of enantiomeric and racemic salts seem to be rather similar to each other [29], which can also be confirmed by IR spectra collection for racemic and enantiomeric HCl salts of ephedrine available in SciFinder^(n)^ [30]. The DSC melting point of our starting material was measured as high as 192.5 °C.

##### Identification of Solid Racemic Chloramphenicol Base Hydrochloride Monohydrate to Be Resolved

**Figure 10:**
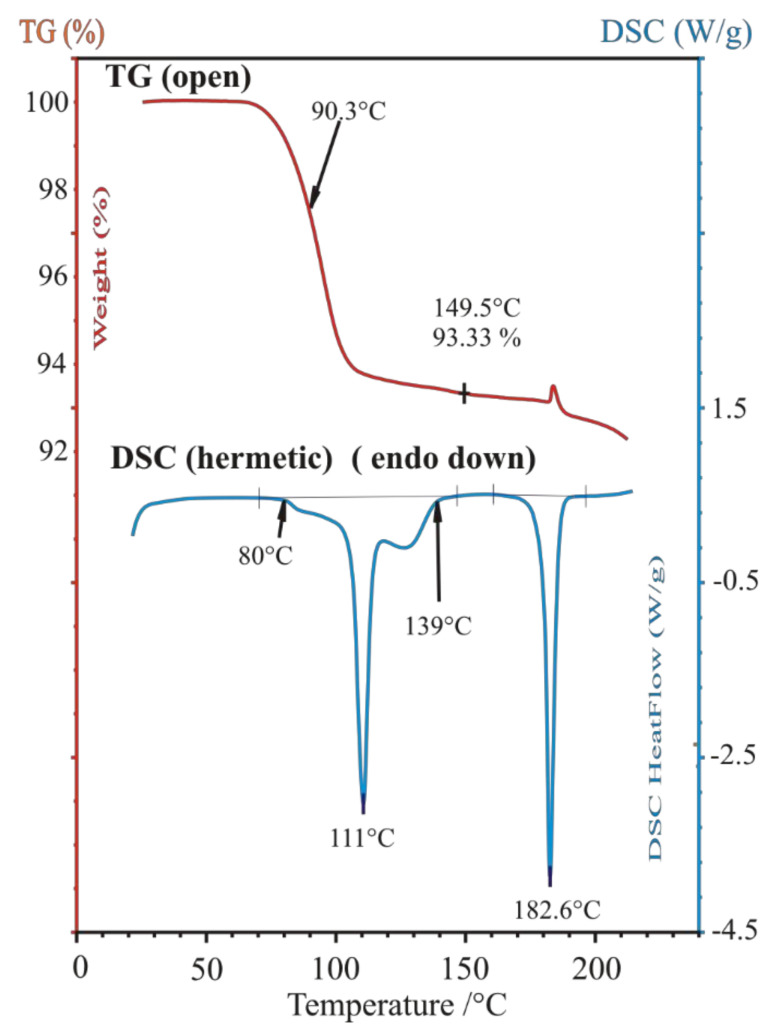
Thermal behavior of racemic Chloramphenicol base hydrochloride monohydrate ‘***rac***-**AD·HCl·H_2_O**’, showing 6.66% loss of ca. one molecule of water of crystallization in open Pt crucible in thermogravimetric (TG) furnace (upper curve), meanwhile heat effects of complex dehydration, dissolution in the water of crystallization, drying and final fusion are observed by DSC in closed Al crucible (lower curve).

The racemic Chloramphenicol base hydrochloride monohydrate sample being resolved, labelled as ‘*rac*-**AD·HCl·H_2_O**’, could not be identified by its powder XRD profile (Figure 7C), as neither the powder pattern reference nor the 3D single-crystal structural coordinates were available for it in the special literature. Nevertheless, it is already mentioned as racemic hydrochloride monohydrate in references. Nos. [32,33,34], as subject to be resolved. No melting point could be observed in the open crucible because of its low-temperature weight loss, which occurred in our sample from 80 °C to 150 °C as a 6.66% loss of the original mass (Figure 10, TG curve, theoretical loss of 1 mol water is 6.76% from C_9_H_12_N_2_O_4_·HCl·H_2_O). Anyhow, by DSC, in a closed Al crucible, at a heating rate of 10 °C/min, we could measure a provisional “melting point” at 110.6 °C during a prolonged loss of water crystallization (Figure 10. DSC curve). The condensed phase of drying material was finally fused at 182.6 °C, probably with decomposition (desamination). The special literature reports the following melting point regions for anhydrous substances: “threo HCl (three times recrystallized from EtOH)” mp. 180–181 °C [35]; “dl-ψ-p-O_2_NC_6_H_4_CH(OH)CH(NH_2_)CH_2_OH HCl salt”, m. 177.5–178.5° [36]; “with an equal part of concentrated HCl to give crystalline 1-p-nitrophenyl-2-amino-1,3-propandiol (II), m. 177–180°” [37]; “dl-threo II-HCl, mp. 179–180 °C” [38].

Calculations to find suitable unit cell parameters of racemic Chloramphenicol base hydrochloride monohydrate ‘***rac***-**AD·HCl·H_2_O**’, based on the actually measured powder XRD pattern by powder pattern indexing ([31]) using interactive DASH program [16], were attempted (Table 4).

##### Identification of Solid Resolving Agents, Monosodium Salts of DBTA and TA

The XRD pattern and FT-IR spectrum of the resolving agent, sodium-dibenzoyl-bitartrate salt, labelled as (-)-**DBBS-Na**, are shown in Figure 7B and Figure 8c, respectively. No reference pattern or spectrum of it is available in the special literature. The XRD pattern and FT-IR spectrum of sodium (*S*,*S*)-bitartrate salt monohydrate, labelled as (*S*,*S*)-**BS-Na·H_2_O,** are exhibited in Figure 7D and Figure 8d. As our preferred revolving co-agent, monohydrated sodium salt of (*S*,*S*)-bitartaric acid, (*S*,*S*)-**BS-Na·H_2_O**, was well identified by either an indexed powder reference of a chiral sodium bitartrate salt monohydrate (PDF-00-031-1303, [19,39] crystallizing in the orthorhombic crystal system, in chiral space group of P2_1_2_1_2_1_ (No. 19), or by a simulated powder pattern using Mercury program [40] from the single-crystal 3D atomic coordinates of Sodium hydrogen L-tartrate monohydrate (CSD refcode of ZZSSS02, [22,41]).

Anhydrous sodium (*R*,*R*)-bitartrate salt, (*R*,*R*)-**BS-Na** (Figure 7E and Figure 8e), could be identified by two simulated powder patterns generated from single-crystal atomic coordinates of anhydrous catena-((μ_6_-hydrogen (+)-tartrato)-sodium), (C_4_H_5_NaO_6_)_n_ (CSD refcodes of YELNIM [42], and YELNIM01 [43]. It could also be prepared/obtained from MeOH.

#### 3.2.3. Characterization of Intermediate Diastereomeric Salts of Resolutions

##### **(1*S*,2*R*)-(+)-Ephedrinium (2*R*,3*R*)-Dibenzoyl-Bitartrate** (1:1 Salt) and Its Solidified Mother Liquor

**Figure 11:**
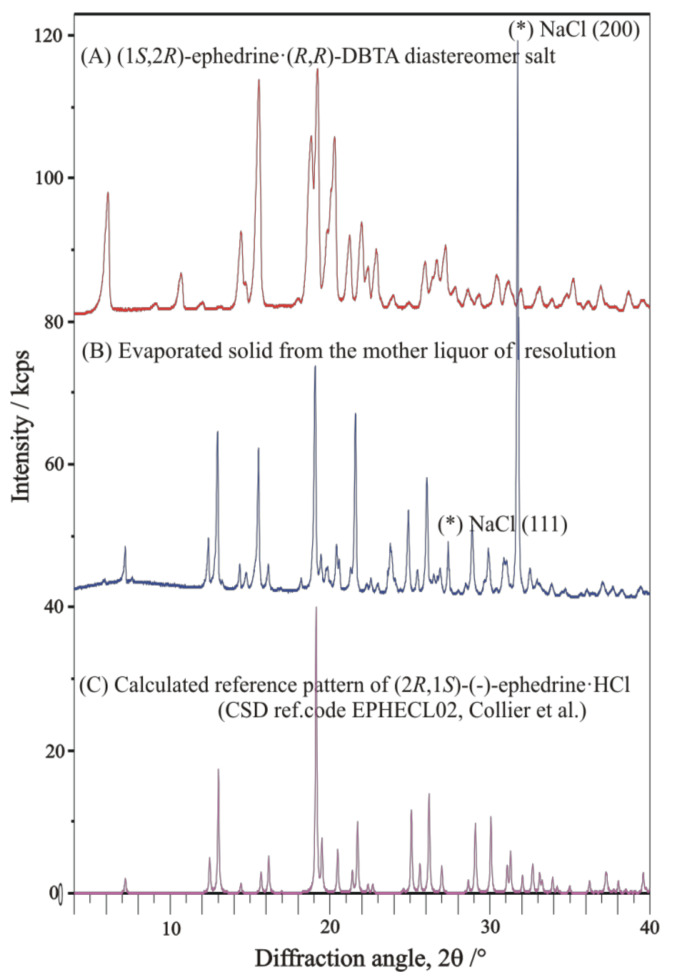
Powder XRD profile of (**A**) diastereomeric salt **I (EPH-DBTA, 1:1),** (**top**); (**B**) crystalline phase (**middle**) obtained by evaporation of mother liquor to dryness, containing mainly NaCl (two peaks at around 2θ = 27.5 and 32°, °, marked by asterisks (*)) and enantiomeric (-)-ephedrine HCl salt identified by (**C**) (**bottom**) simulated powder pattern generated from EPHECL02 single crystal structure determination [24]. Some small but unidentified peaks of the middle profile might come from the other member of the diastereomeric salt pair.

**(1*S*,2*R*)-(+)-Ephedrinium (2*R*,3*R*)-dibenzoyl-bitartrate** (1:1 salt, its molar ratio is established by measurement of N-content), labelled as ‘**I, EPH-DBTA (1:1)**’, was formed during the resolution process of racemic ephedrine hydrochloride with half an equivalent amount of (2*R*,3*R*)-dibenzoyl-bitartaric acid sodium salt in an almost quantitative yield and very high diastereomeric access. Meanwhile, sodium chloride and the hydrochloride salt of the other enantiomeric (1*R*,2*S*)-(-)-ephedrine remained in the mother liqour. After the evaporation of the mother liqor, both of these two crystalline by-products could be identified by XRD (Figure 11), the latter one with the help of generated powder patterns of either EPHECL [25], EPHECL01 [26], EPHECL02 [24], or EPHECL05 [27] from single-crystal data collected in CSD. The corresponding reaction equation can be formulated as the following:

*rac*-EPH·HCl + 0.5 (2*R*,3*R*)-dibenzoylbitartrate-Na = 0.5 NaCl + 0.5 (1*S*,2*R*)-(-)-EPH·HCl + 0.5 (1*R*,2*S*)-(+)-EPH·(2*R*,3*R*)-dibenzoylbitartrate (1:1) salt (**I**)

**Figure 12:**
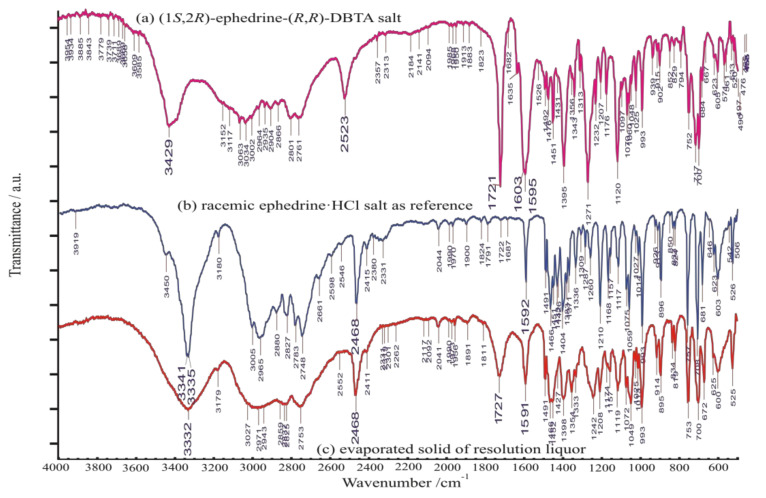
FT-IR spectrum of (**a**) diastereomeric salt ‘**I (EPH-DBTA,1:1)**’ (**top**) and the (**c**) solid phase obtained by evaporation of mother liquor to dryness (**bottom**), containing mainly enantiomeric (-)- ephedrine HCl salt identified and represented by (**b**) (**middle**) the rather similar spectrum [29,30] of starting racemic ephedrine hydrochloride salt to be resolved. Significant absorption of ν(C=O) of the bottom spectra at 1727 cm^−1^ might come from the other (minor) member of the diastereomeric salt pair.

The XRD pattern and FT-IR spectrum of ‘**I, EPH-DBTA (1:1)**’ salt are shown in Figure 11A and Figure 12a, while that of evaporated residues are shown in Figure 11B and Figure 12c, respectively. The FT-IR spectrum of solid obtained by evaporation to dryness of the corresponding mother liquor (Figure 12c) resembled mainly the ephedrine HCl-salt(s), with some indication on the presence of the other diastereomeric salts, as well.

**Figure 13:**
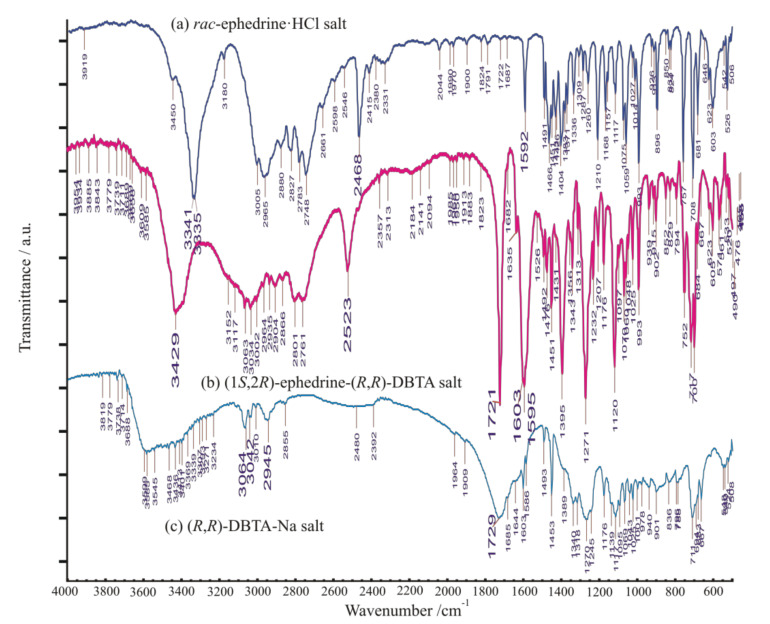
FT-IR spectrum of (**a**) starting racemic ephedrine hydrochloride salt (‘***rac*-EPH·HCl**’) to be resolved (top); (**b**) diastereomeric salt ‘**I (EPH-DBTA, 1:1)**’, (middle); (**c**) starting enantiomeric sodium (*R*,*R*)-bitartrate salt monohydrate, ‘**(*R,R*)-BS-Na·H_2_O**’ (bottom).

Both XRD pattern and the FT-IR spectrum of precipitated diastereomeric salt is quite different from that of its starting materials (Figure 7 and Figure 13), indicating firmly formation of a definitely new crystalline solid substance.

**Figure 14:**
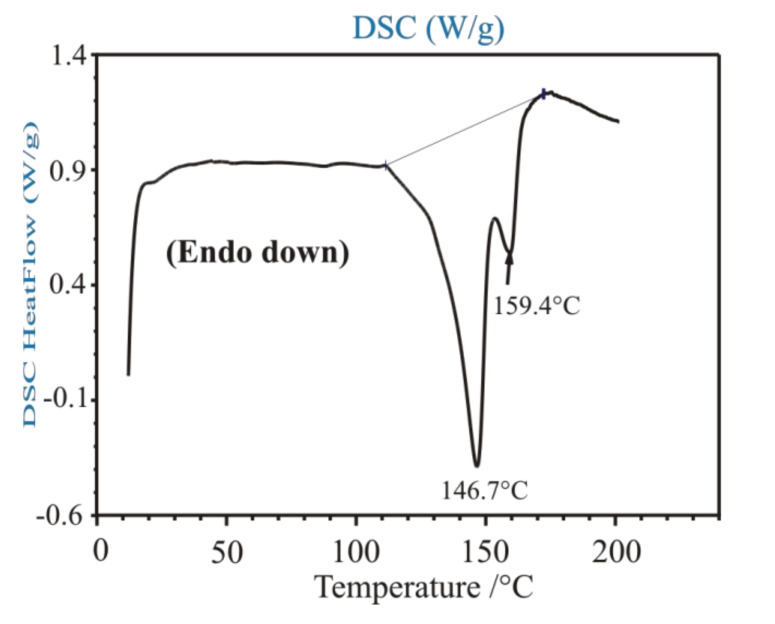
DSC curve of the ‘**I (EPH-DBTA)**’ diastereomeric salt. Unfortunately, it did not show any definite melting point by DSC. It only showed two elongated endothermic heat effects indicating a probable reaction between its ammonium and carboxylate components as a partial amid formation (DSC).

Unfortunately, the ‘**I (EPH-DBTA)**’ salt did not show any definite melting point by DSC (Figure 14). It only showed elongated endothermic heat effects, probably indicating a reaction between its ammonium and carboxylate components as a partial amid formation (DSC), already at moderately high temperatures, over 100 °C.

##### Diastereomeric Solids from Resolution Experiments of Racemic Chloramphenicol Base

**Figure 15:**
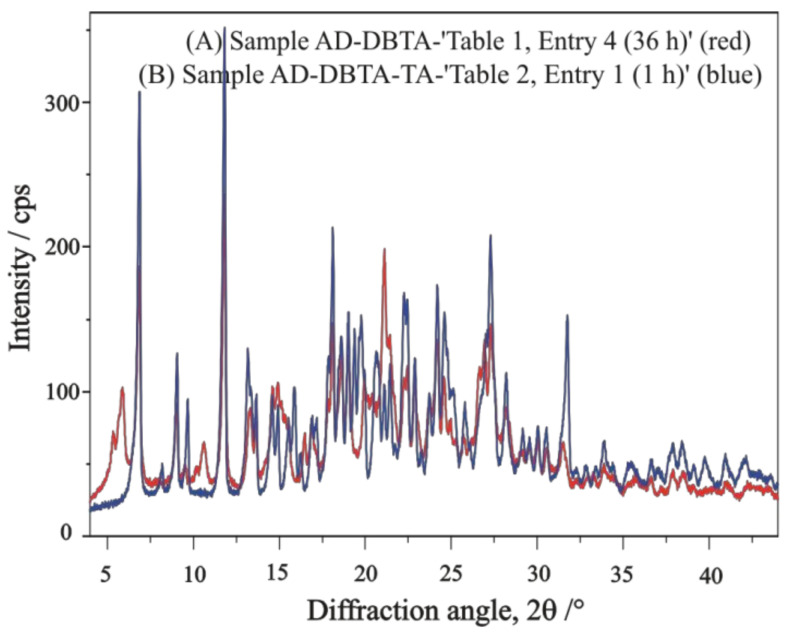
XRD profiles of ‘**AD-DBTA**’ diastereomeric samples obtained in resolution trials of ‘Table 1, Entry 4 (36 h)’ (red) and ‘Table 2, Entry 1 (1 h)’ (blue) without and with “quasi racemic”, i.e., enantiomeric tartaric acid Na salt as an additive.

**Figure 16:**
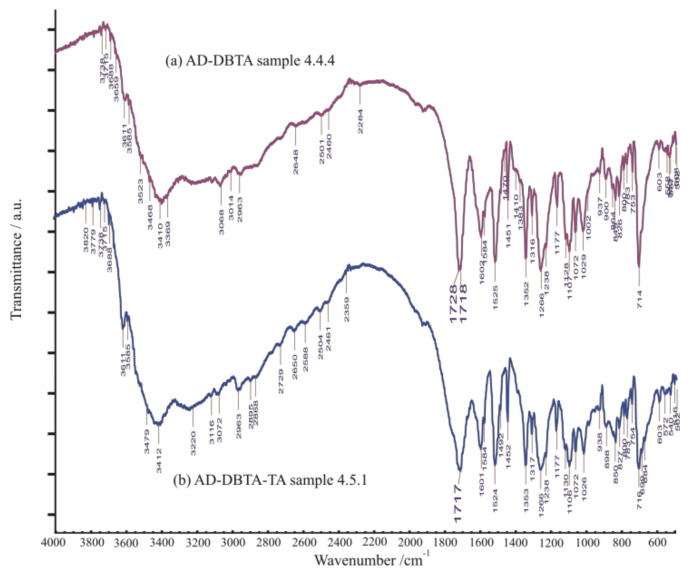
FT-IR spectra of diastereomeric samples obtained in resolution trials of Sample ‘Table 1, Entry 4 (36 h)’ (**top**) and Sample ‘Table 2, Entry 1 (1 h)’ (**bottom**) without and with “quasi racemic”, i.e., enantiomeric tartaric acid Na salt as an additive. Unfortunately, they resemble each other. There might be some indication of the presence of the **TA-Na**-salt additive in the case of Sample ‘Table 2, Entry 1 (1 h)’ (**bottom**).

The XRD profile of Sample ‘Table 1, Entry 4 (36 h)’ probably partially contains the other pair of diastereomeric salts precipitated simultaneously if compared with the profile of Sample ‘Table 2, Entry 1 (1 h)’ (Figure 15). In contrast, the FT-IR spectrum of Sample ‘Table 2, Entry 1 (1 h)’, in comparison with that of Sample ‘Table 1, Entry 4 (36 h)’, is suspected of containing some of the initial compounds in their initial forms (Figure 16), especially sodium bitartrate co-resolving agent, promoting a kind of quick co-precipitation process of solid phases present in the system.

##### Unsuccessful Trials of Resolution of EPH and AD with Single Sodium Bitartrate Salt as Resolving Agent

**Figure 17:**
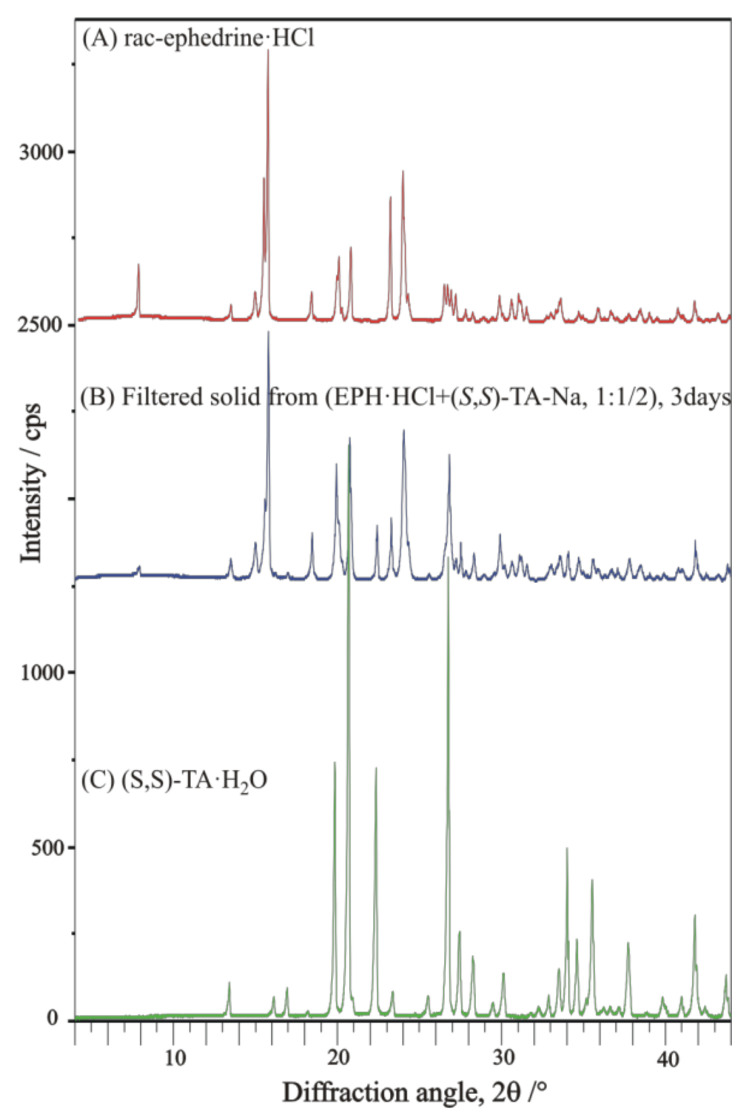
XRD profile of filtered solid from resolution trial of ***rac*-EPH-HCl** salt with enantiomeric **TA-Na**-salt, as a single resolving agent, in comparison with that of initial components (***rac***-**EPH-HCl** salt and **TA-Na-H_2_O**-salt).

The resolution trial of ***rac***-**EPH-HCl** salt with enantiomeric **TA-Na**-salt as a single resolving agent was not successful and resulted in the solidification of the original components (***rac***-**EPH-HCl** salt and **TA-Na-H_2_O**-salt), as indicated by the XRD profile comparison of the filtered final solid with that of the initial chemicals (Figure 17).

**Figure 18:**
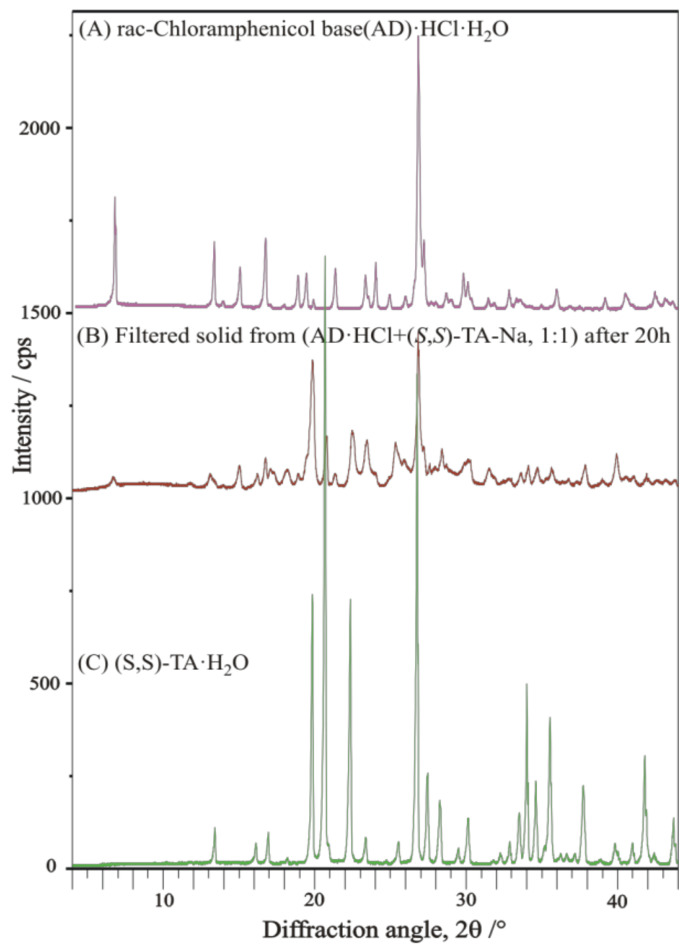
XRD profile of filtered solid from resolution trial of ***rac***-**AD-HCl-H_2_O** salt with enantiomeric **TA-Na**-salt as a single resolving agent, in comparison with that of initial components (***rac***-**AD-HCl-H_2_O** salt and **TA-Na-H_2_O**-salt).

The resolution trial of ***rac***-**AD-HCl-H_2_O** salt with enantiomeric **TA-Na**-salt as a single resolving agent was not successful and resulted in the solidification of the original components (***rac***-**AD-HCl-H_2_O** salt and **TA-Na-H_2_O**-salt), as indicated by the XRD profile comparison of filtered final solid with that of initial chemicals (Figure 18).

##### Repeated Trials of Resolution of EPH·HCl with (*R*,*R*)-DBTA-Na and Sodium Bitartrate Salt as Quasi-Enantiomeric Resolving Agent-Mixture

**Figure 19:**
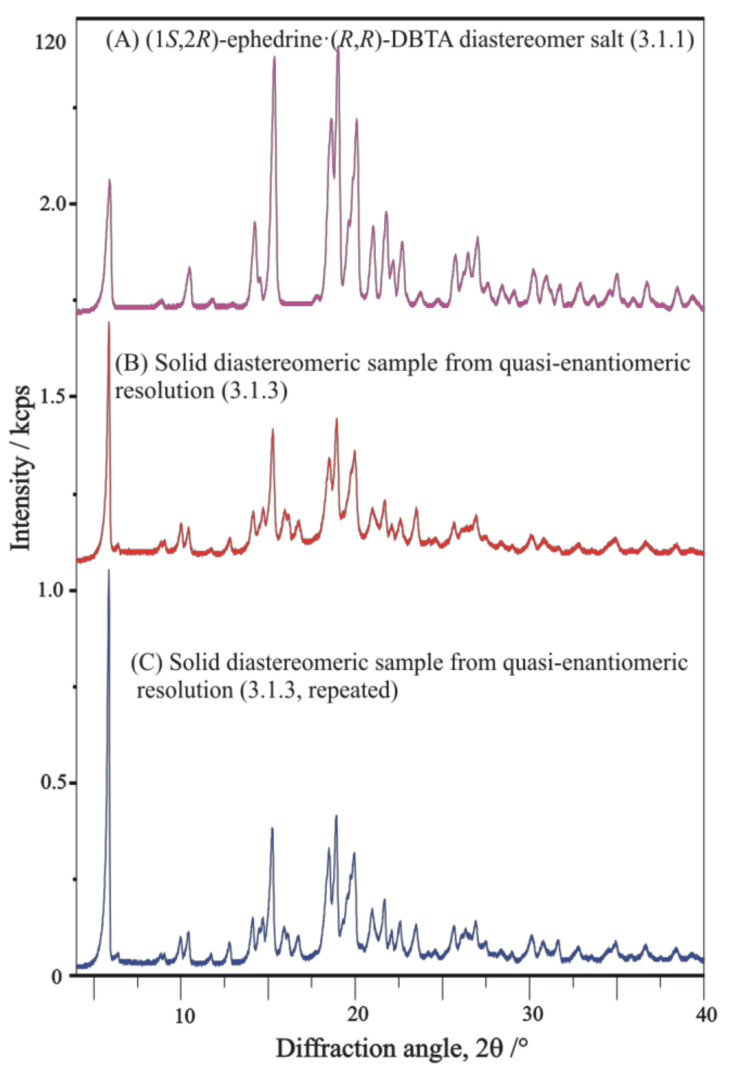
XRD profiles of solid diastereomeric products in resolution trials of racemic **EPH·HCl** applying (*R*,*R*)-**DBTA-Na** without (**A**) and with (**B**,**C**) (*R*,*R*)-**TA-Na** quasi-enantiomeric resolving co-agent. The XRD profiles of the obtained diastereomeric salt samples are proved to be rather similar to each other, indicating that the diastereomeric intermediate salt of the quasi-enantiomeric resolutions contain only **DBTA**, while enantiomers of **TA** are not involved in the composition of diastereomeric salt samples, and no occurrence of the opposite enantiomeric EPH can be expected at all.

The resolution trials of racemic **EPH·HCl** with (*R*,*R*)-**DBTA** and (*R*,*R*)-**TA-Na** together, i.e., with a quasi-enantiomeric resolving agent mixture, resulted in a similar XRD profile of final solid products (Figure 19). That means the diastereomeric salts samples of the quasi-enantiomeric resolutions contain only **DBTA;** any enantiomers of **TA** are not involved in the stoichiometry of diastereomeric salts. Additionally, it indicates that, in this particulate case, no occurrence of the other enantiomeric **EPH** with opposite chirality can be expected at all.

## 4. Conclusions

The resolution of racemic ephedrine·HCl using the enantiomers of tartaric acid (**TA**) and dibenzoyltartaric acid (**DBTA**) as resolving agents was attempted, of which the **DBTA** enantiomers proved to be favorable. It was found that by using 0.5 molar equivalent of (2*R*,3*R*)-**DBTA** with a good yield (92.5%) and enantiomeric purity (ee ~100%), the (1*S*,2*R*)-(+)-**EPH** enantiomer can be isolated (F: 0.92) from the precipitated diastereomeric salt.

The resolution of racemic **EPH·HCl** was investigated with a 1:1 quasi-enantiomeric resolving agent mixture of (2*R*,3*R*)-**DBTA** and (*R*,*R*)-**TA** in acetone and water. From the aqueous solution after the dissolution of the resolving agent mixture ((2*R*,3*R*)-**DBTA·Na** and (*R,R*)-**TA·Na** salts), the diastereomeric salt was immediately precipitated, from which the (1*S*,2*R*)-(+)-**EPH**) enantiomer was separated with a good yield (87.5%) and high enantiomeric purity (90.2%). Thus, during the resolution of racemic **EPH·HCl** with 0.5 mol of (2*R*,3*R*)-**DBTA** from water, the (1*S*,2*R*)-(+)-**EPH** and from a quasi-racemic solution containing 0.5 mol of (*R*,*R*)-**TA**, the same **EPH** enantiomer immediately formed diastereomeric salt. In conclusion, when the resolving agent and its quasi-enantiomeric mixture were used from the same solvent, identical enantiomers crystallized. Using the quasi-racemic and quasi-enantiomeric resolving agents did not produce the expected results, as in the case of the resolution of racemic Tofisopam [12]; however, it did assist in the precipitation of diastereomeric salts, whether they were desalted due to increased ionic strength (“salt effect”) or participated in the diastereomeric salt precipitation as nucleating agents, by showing quick, almost kinetic resolution.

**Figure 20:**
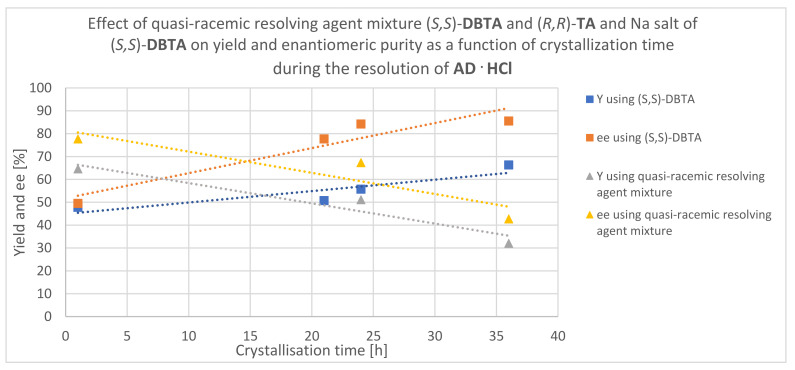
In the case of the use of the quasi-racemic and single enantiomeric resolving agent, in one case, the effect of kinetic and in the other, the effect of thermodynamic control prevails during the resolution of **AD·HCl**.

During the resolution of **AD·HCl** with 0.5 equivalents of (*S*,*S*)-**DBTA·Na**, it was found that the effect of thermodynamic control prevailed, so for the best separation, the thermodynamic equilibrium must be waited for. However, during the resolution of **AD·HCl** with a quasi-racemic resolving agent, the effect of the kinetic control prevails (Figure 20), and a better result can be obtained using this method than with 0.5 equivalents of (*S*,*S*)-**DBTA·Na**.

Meanwhile, the effects were followed by comprehensive analytical (XRD, FTIR, and DSC) examinations, the crystallographic unit cell of initial racemic hydrochloric salts were attempted to refine or establish, applying the powder XRD pattern-indexing opportunities connected to the DASH software package.

## Figures and Tables

**Table 1:** Results for the Aminodiol enantiomer obtained from the diastereomeric salt and the mother liquor using (*S*,*S*)-**DBTA·Na**.

Entry	Crystallization Time [h]	Resolving Agent ^a^	Y ^b^ [%]	ee ^c^ [%]	F ^c^ [-]	Y ^b^ [%]	ee [%]	F ^c^ [-]
			from Diastereomer			from Mother Liquor		
1	1	(*S*,*S*)-**DBTA·Na**	47.8	49.5	0.24	44.7	11	0.05
2	21	(*S*,*S*)-**DBTA·Na**	50.7	77.7	0.39	56.3	17.6	0.1
3	24	(*S*,*S*)-**DBTA·Na**	55.7	84.2	0.47	54.6	28.9	0.16
4	36	(*S*,*S*)-**DBTA·Na**	66.3	85.5	0.57	84.8	50.7	0.43

^a^ applied in half-equivalent amounts. ^b^ Based on the half of the racemate that is deemed 100% for each antipode. ^c^ Resolving capability (resolvability), also known as the Fogassy-parameter [F = (Yield/100) × (ee/100)].

**Table 2:** Effect of crystallization time using the quasi-racemic resolving agent mixture on yield and enantiomeric purity measured from the enantiomers obtained from the diastereomeric salt.

Entry	Crystallization Time [h]	Quasi-Racemic Resolving Agent Mixture ^a^	Y ^b^ [%]	Ee ^b^ [%]	F ^c^ [-]	Y ^b^ [%]	Ee ^b^ [%]	F ^c^ [-]
			from Diastereomer			from Mother Liquor		
1	1	(*S*,*S*)-**DBTA·Na** (*R*,*R*)-**TA·Na**	71.2	97.7	0.7	64.6	77.7	0.5
2	24	(*S*,*S*)-**DBTA·Na** (*R*,*R*)-**TA·Na**	64.1	77.4	0.5	51.1	67.3	0.34
3	36	(*S*,*S*)-**DBTA·Na** (*R*,*R*)-**TA·Na**	52.2	64.5	0.34	32	42.7	0.14

^a^ applied in an equivalent amount. ^b^ Based on the half of the racemate that is deemed 100% for each antipode. ^c^ Resolving capability (resolvability), also known as the Fogassy-parameter [F = (Yield/100) × (ee/100)].

**Table 3:** Former unit cell parameters of ephedrine hydrochloride and that of ‘*rac*-**EPH·HCl**’ sample estimated from the measured powder XRD patterns by powder pattern indexing (Dicvol [31]) using the interactive DASH program [16].

Crystallographic Unit Cell Parameters	According to [20]	PDF 00-032-1675 [18]	Structural Trial from Powder Profile [DASH, This Work]
crystal system	monoclinic		
space group	P21/a (No.14)		
a (Å)	13.44	13.405	13.376
b (Å)	7.04	7.040	7.036
c (Å)	13.27	13.242	13.230
α (°)	90		
β (°)	118.40	118.54	118.69
γ (°)	90		
V (Å^3^)	1104.465	1097.81	1092.32
Z/Z’	4/1		
Vm (Formula unit, Å^3^)	276.116	274.452	273.08
zero-point shift (°)	n.a	n.a	0.108

**Table 4:** First guesses for unit cell parameters of racemic Chloramphenicol base hydrochloride monohydrate ‘*rac*-**AD·HCl·H_2_O**’ estimated from the measured powder XRD patterns by powder pattern indexing ([31]) using the interactive DASH program [16].

Crystallographic Unit Cell Parameters	Structural Guess from Powder Profile by DASH [16], This Work
crystal system	triclinic
space group	*P*-1, No. 2
a (Å)	13.12
b (Å)	7.279
c (Å)	6.940
α (°)	113.8
Β (°)	99.54
γ (°)	87.61
V (Å^3^)	597.71
Z/Z’	2/1
V_m_ (Formula unit, Å^3^)	298.855
zero-point shift (°)	0.091

## Data Availability

Not applicable.

## Sample Availability

Samples of the compounds are available from the authors.
